# Shelby Medical College, Nashville, Tenn.

**Published:** 1858-07

**Authors:** 


☞ We have received the following Circular, which we take pleasure in inserting, and at the same time, in expressing our cordial good will towards the movement, and desire for its eminent success.

We are in favor of the largest liberty, and the most full and free offering of medical advantages throughout the country, and should the plan be adopted, of which we have been an advocate for a length of time, and a recommendation of which is contained in the late report upon Medical Education to the American Medical Association, viz: *The separation of the Teaching and Diploma-granting power*, every vestige of objection to the multiplication of Medical Schools, will have been removed. All we desire for ourselves, is a fair field, and to be judged by our *merits*, and this we are more than willing to grant to others.

### Shelby Medical College,

### Nashville, Tenn.

### FIRST ANNUAL COURSE OF LECTURES.

The preliminary course of Lectures in this Institution will commence on the first Monday in October, 1858, and extend through the month. It is advisable that Students, who contemplate attending through the winter, should commence with the Preliminary Lectures.

The Regular Course will open on Monday, the first day of November, 1858, and continue to the 1st of March ensuing, and will comprise full lectures on

General and Microscopical Anatomy;
Physiology and Pathology;
Chemistry and Pharmacy;
Materia Medica and Therapeutics;
Theory and Practice of Medicine;
Obstetrics, and diseases of Women and Children, and
Principles and Operations of Surgery.

*Surgical and Medical Clinics* will be given twice weekly, and will constitute an important feature in the course of instruction.

The new and commodious College Buildings, with Hospital attached, are located on the corner of Broad and Vine streets.

The Anatomical Rooms, under the direction of the Professor of Anatomy, will be opened on the first Monday in October, and an ample supply of the best material will be afforded through the Winter.

*Regular announcements, with details of the Faculty organization, will be issued in a few weeks.*

For further information address

JOHN P. FORD, M. D.,
*Dean of the Faculty.*

Nashville, Tenn., June 7th, 1858.

